# Suboptimal infant and young child feeding practices among internally displaced persons during conflict in eastern Ukraine

**DOI:** 10.1017/S1368980017003421

**Published:** 2017-12-22

**Authors:** Aimee Summers, Oleg O Bilukha

**Affiliations:** 1 Epidemic Intelligence Service, Centers for Disease Control and Prevention, Atlanta, GA, USA; 2 Emergency Response and Recovery Branch, Division of Global Health Protection, Center for Global Health, Centers for Disease Control and Prevention, 1600 Clifton Road, Mailstop E-22, Atlanta, GA 30329, USA

**Keywords:** Infant and young child feeding, Breast-feeding, Bottle-feeding, Ukraine, Conflict, Surveys and questionnaires, Child health

## Abstract

**Objective:**

To determine current status, areas for improvement and effect of conflict on infant and young child feeding (IYCF) practices among internally displaced persons (IDP) in eastern Ukraine.

**Design:**

Cross-sectional household survey, June 2015.

**Setting:**

Kharkiv, Dnipropetrovsk and Zaporizhia *oblasts* (Ukrainian administrative divisions) bordering conflict area in Ukraine.

**Subjects:**

Randomly selected IDP households with children aged <2 years registered with local non-governmental organizations. Questions based on the WHO IYCF assessment questionnaire were asked for 477 children. Mid-upper arm circumference was measured in 411 children aged 6–23 months.

**Results:**

Exclusive breast-feeding prevalence for infants aged <6 months was 25·8 (95 % CI 15·8, 38·0) %. Percentage of mothers continuing breast-feeding when their child was aged 1 and 2 years was 53·5 (95 % CI 43·2, 63·6) % and 20·6 (95 % CI 11·5, 32·7) %, respectively. Bottle-feeding was common for children aged <2 years (68·1 %; 95 % CI 63·7, 72·3 %). Almost all infants aged 6–8 months received solid foods (98·6 %; 95 % CI 88·5, 99·9 %). Mothers who discontinued breast-feeding before their infant was 6 months old more often listed stress related to conflict as their primary reason for discontinuation (45·7 %) compared with mothers who discontinued breast-feeding when their child was aged 6–23 months (14·3 %; *P*<0·0001).

**Conclusions:**

To mitigate the effects of conflict and improve child health, humanitarian action is needed focused on helping mothers cope with stress related to conflict and displacement while supporting women to adhere to recommended IYCF practices if possible and providing appropriate support to women when adherence is not feasible.

Appropriate infant and young child feeding (IYCF) practices have a large impact on child survival. Optimum breast-feeding practices alone could prevent an estimated 1·4 million deaths of children under 5 years of age worldwide^(^ ^1^ ^)^. Specifically, exclusive breast-feeding until 6 months of age and continued breast-feeding up to 1 year of age have the greatest impact on reducing child mortality and prevent many deaths from diarrhoea, pneumonia and malnutrition^(^ ^1^ ^–^ ^4^ ^)^. Furthermore, early initiation of breast-feeding prevents neonatal morbidity and mortality^(^ ^5^ ^–^ ^7^ ^)^. In addition to lower mortality from infection and malnutrition, breast-fed children also have a lower risk of dying from sudden infant death syndrome compared with children who are not breast-fed^(^ ^1^ ^,^ ^8^ ^–^ ^10^ ^)^.

IYCF practices beyond breast-feeding also have a large impact on reducing child mortality. For instance, introducing complementary foods at 6 months of age is the third leading child health intervention in preventing child deaths^(^ ^4^ ^)^. Due to the evidence of child health benefits, the WHO and UNICEF recommend early initiation of breast-feeding within the first hour of birth, exclusive breast-feeding for the first 6 months of life, continued breast-feeding until 2 years of age, and timely introduction of nutritious complementary foods at 6 months of age^(^ ^11^ ^)^.

Despite these recommendations, many women, including those in high- and middle-income countries, do not follow the recommended IYCF practices. For example, only two-thirds of infants in the USA are breast-fed within an hour of birth and less than a third of infants are exclusively breast-fed until 6 months of age or are continuing to be breast-fed until 1 year of age, according to national surveys conducted by the Centers for Disease Control and Prevention (CDC)^(^ ^12^ ^)^. These findings are similar to those from middle-income countries in Eastern Europe bordering Ukraine, including Belarus, where in 2012 only about half of infants were breast-fed within the first hour of birth, one-fifth of infants were exclusively breast-fed until 6 months of age and less than one-third of infants continued to be breast-fed at 1 year of age according to Multiple-Indicator Cluster Survey (MICS) data^(^ ^13^ ^)^. IYCF practices were similarly poor in eastern Ukraine. In 2012 the Ukrainian MICS found that only 62 % of infants in the eastern region of Ukraine were breast-fed within an hour of birth and 21 % exclusively breast-fed until 6 months of age^(^ ^14^ ^)^.

Prior to the beginning of the conflict in February 2014, Ukraine was considered a stable, middle-income country. Hostilities in the eastern *oblasts* (administrative divisions of Ukraine) of Donetsk and Luhansk began in June 2014. The conflict has caused political and financial instability and mass displacement. As of June 2015, there were a total of 1·4 million internally displaced persons (IDP) and over 5 million people affected by the conflict^(^ ^15^ ^)^. At that time, the majority of IDP lived in Donetsk and Luhansk *oblasts* (conflict zone) and in Kharkiv, Dnipropetrovsk and Zaporizhia *oblasts* bordering the conflict zone. The influence of this conflict on IYCF practices among the affected population was unknown, but important to consider. Complex humanitarian emergencies can lead to suboptimal hygiene and health-care practices, especially considering that overcrowding is common as IDP move into collective centres and homes of friends and relatives^(^ ^16^ ^)^. Furthermore, nutritious complementary foods may be unavailable and water sources may be compromised during complex humanitarian emergencies^(^ ^17^ ^)^. This can lead to a high risk of diarrhoea and infections, making optimal IYCF practices such as exclusive breast-feeding in infants until 6 months of age even more essential^(^ ^16^ ^)^. In addition, it is important that suboptimal feeding practices be identified, managed and supported appropriately if approaches such as artificial feeding are warranted. Given Ukraine’s suboptimal pre-crisis IYCF practices, families with infants and young children affected by the conflict were of particular concern. The present study aimed to describe the current status, areas for improvement and effect of conflict on IYCF practices of IDP in Kharkiv, Dnipropetrovsk and Zaporizhia *oblasts* bordering the conflict zone.

## Methods

We conducted a cross-sectional household survey among IDP households with children less than 2 years of age residing in Kharkiv, Dnipropetrovsk and Zaporizhia *oblasts*. The total sample size of 474 children was determined based on an expected 50 % prevalence of current breast-feeding and a ±4·5 % precision^(^ ^14^ ^)^. *Oblast*-specific sample sizes, allocated based on the relative numbers of IDP living in each *oblast*, were 230, 130 and 114 children from Kharkiv, Dnipropetrovsk and Zaporizhia, respectively.

We obtained lists of registered IDP families with infants and young children from humanitarian aid agencies to create the sampling frame. Lists were checked for duplicates and merged. A household was defined as persons living under the same roof who were registered together to receive assistance. The following criteria were used to identify IDP households eligible for the study: (i) household was included in at least one humanitarian agency list received; (ii) a working telephone number was provided on the registration list and a household could be reached by telephone; (iii) children less than 2 years old were living in the household; (iv) household was residing in Kharkiv, Dnipropetrovsk or Zaporizhia *oblast* at the time of the survey; and (v) the mother/caregiver consented to participate.

We independently selected households for each *oblast*. Lists were randomized and households were contacted in that random order via telephone until the required number of consenting households was reached. During the initial contact, we verified the child’s age and whether the household was residing in the survey area. If the household did not meet the inclusion criteria or a household could not be reached after three call attempts on the day of the scheduled survey team visit, that household was removed from the list and the next household on the list was called. For households meeting the inclusion criteria during telephone screening, an in-person interview visit was scheduled. All children less than 2 years of age present in the household during the in-person interview were included in the survey.

The survey included questions about the child (age, sex), mother (age, education, total number of children) and household (current location, living situation, permanent residence before displacement, length of displacement, number of people living in the household, total number of children less than 2 years of age living in the household, sex of the head of household, current household employment and whether the household received humanitarian assistance). Questions on IYCF practices, derived from the standard WHO IYCF questionnaire, were also included^(^ ^18^ ^)^. The questionnaire was translated into Russian and the accuracy of the translation was verified by Russian-speaking CDC staff.

Questionnaires were administered to the child’s mother, unless she was unavailable. If the mother was unavailable, the questionnaire was administered to a second caregiver with knowledge of the child’s feeding practices. Mid-upper arm circumference (MUAC) was measured following standard procedures in each child aged 6–23 months. MUAC<115 mm was classified as severe acute malnutrition, MUAC=115–124 mm as moderate acute malnutrition and MUAC≥125 mm as no malnutrition^(^ ^19^ ^,^ ^20^ ^)^. Verbal informed consent of the respondent was obtained prior to administration of the questionnaire.

Household survey questionnaires were administered by trained enumerators located in eastern Ukraine with experience in conducting household surveys. Enumerators were trained by Russian-speaking CDC staff on sampling procedures, questionnaire administration, interview techniques, telephone screening procedures to identify eligible households and MUAC measurement techniques. A standardization exercise on measuring MUAC in children included ten children aged 6–23 months who were ineligible for the survey. Technical error of measurement and bias were calculated for each enumerator to assess accuracy and precision of measurements^(^ ^21^ ^)^.

Data were entered in Epi Info version 7.1.5.0. Double data entry was performed and discrepancies were reconciled using the original paper form. All analyses were conducted using the statistical software package STATA version 13 and Microsoft^®^ Excel 2013. WHO IYCF indicators were calculated according to the instructions in the WHO manual^(^ ^18^ ^)^. The dietary diversity indicator in the present study was measured using six food groups instead of the standard seven food groups used by WHO, by combining vitamin-A rich fruits and vegetables with other fruits and vegetables, as it was more appropriate to the Ukrainian context. Due to fewer food groups, the proportion of children 6–23 months of age receiving foods from three or more food groups (instead of the WHO standard four or more foods groups) was considered adequate minimum dietary diversity in the present study^(^ ^18^ ^)^. Children’s complementary feeding practices were assessed by calculating the proportions of the types of foods and liquids children consumed in the 24 h preceding the survey and the mean age at which various foods and liquids were introduced.

Using bivariate and multivariate logistic regression models, we assessed the association of potential risk factors with key IYCF indicators as the outcome variables in order to identify target populations for improvement of IYCF practices. Potential risk factors used as predictor variables were: child’s age and sex, whether the child was born before or after displacement, maternal age and education level, number of children born to the mother, current location of the household, length of displacement, total number of children aged less than 2 years living in the household, sex of the head of household, place of permanent residence before displacement, whether the household was paying rent and current household employment. We assessed eight outcome variables, all key IYCF indicators: whether the child was ever breast-fed, early initiation of breast-feeding within an hour of birth, exclusive breast-feeding of infants aged less than 6 months, continued breast-feeding at 1 and 2 years of age, introduction of solid foods, bottle-feeding and minimum meal frequency. Each outcome variable was tested in bivariate analyses with each potential risk factor. Statistically significant risk factors at a *P*≤0·05 level were included in a multivariate logistic regression model to estimate OR and 95 % CI. If no risk factors were found to be statistically significant in the bivariate analyses, a multivariate model was not constructed. Data presented in the results are from the multivariate models.

## Results

The household survey was conducted between 8 June 2015 and 19 June 2015. A total of 2278 households were contacted by telephone and 770 (33·8 %) were eligible. Data were collected on 458 eligible households resulting in a response rate of 59·5 % (458/770). Table 1 shows the eligibility, reasons for non-eligibility and response rate for eligible households among internally displaced households contacted via telephone for the survey.

**Table 1:** Eligibility, reasons for non-eligibility and response rate among internally displaced households residing in Kharkiv, Dnipropetrovsk and Zaporizhia *oblasts*, eastern Ukraine, contacted via telephone during June 2015

	Ineligible households (*N* 1508)							Eligible households (*N* 770)				
	Not reached by phone		Moved		Child aged ≥2 years		Total ineligible	Refused		Consented		Total eligible
Oblast	*n*	%	*n*	%	*n*	%	*n*	*n*	%	*n*	%	*n*
Dnipropetrovsk (*N* 886)	246	37·3	182	27·6	231	35·1	659	104	45·8	123	54·2	227
Zaporizhia (*N* 409)	98	37·1	84	31·8	82	31·1	264	32	22·1	113	77·9	145
Kharkiv (*N* 983)	198	33·9	199	34·0	188	32·1	585	176	44·2	222	55·8	398
Total (*N* 2278)	542	36·0	465	30·8	501	33·2	1508	312	40·5	458	59·5	770

There were 230, 130 and 117 children surveyed in Kharkiv, Dnipropetrovsk and Zaporizhia *oblasts*, respectively. Child, maternal and household characteristics are shown in Table 2. Among the 477 children, 51·8 % were male and the mean age was 12·8 (sd 5·8) months. Of the 458 mothers surveyed, the mean age was 30·1 (sd 5·3) years, 59·6 % had completed a minimum of a college degree and 43·0 % only had one child. The majority of the 458 internally displaced households were located in the *oblast* centre (75·8 %). Most households (76·0 %) were responsible for paying rent for their current dwelling. Only forty-two households (9·2 %) had been displaced for less than 6 months, 289 (63·1 %) had been displaced for between 6 and 11 months, and 127 (27·7 %) had been displaced for 1 year or longer. Almost all households (94·3 %) had only one child aged less than 2 years living in the household. The mean number of people living in a household was 4·2 (sd 1·6).

**Table 2:** Child, maternal and household characteristics among internally displaced persons residing in Kharkiv, Dnipropetrovsk and Zaporizhia *oblasts*, eastern Ukraine, June 2015

Characteristic	*n*	%
Child (*N* 477)		
Male	247	51·8
Age (months)		
0–5	66	13·8
6–11	163	34·2
12–17	152	31·9
18–23	96	20·1
Maternal (*N* 458)		
Age (years)		
<25	64	14·0
25–29	157	34·3
30–34	151	33·0
≥35	86	18·8
Completed higher education	273	59·6
Total number of children		
1	197	43·0
≥2	261	57·0
Household (*N* 458)		
Household location		
*Oblast* center	347	75·8
Other city or village	111	24·2
Living situation		
Renting an apartment or house	348	76·0
Living with relatives or friends (no fee)	78	17·0
Collective centre (no fee)	31	6·8
Other	1	0·2
*Oblast* of origin		
Donetsk	289	63·1
Luhansk	162	35·4
Other	7	1·5
Length of displacement		
<6 months	42	9·2
6–11 months	289	63·1
≥12 months	127	27·7
Total no. of children aged <2 years in household		
1	432	94·3
2	26	5·7
Head of household		
Male	229	50·0
Female	223	48·7
Don’t know	6	1·3
Resident of household earning money	245	53·5

### Ever breast-fed

Table 3 shows the prevalence of key WHO IYCF indicators in our survey population. The majority of children were ever breast-fed (93·3 %; 95 % CI 90·7, 95·2 %), which was similar among children born during the conflict in the 11 months preceding the survey (93·9 %; 95 % CI 90·0, 96·6 %) and those born before the conflict began in the 12–23 months preceding the survey (92·7 %; 95 % CI 88·8, 95·6 %). Among infants aged <6 months, 7·6 (95 % CI 2·5, 16·8) % were never breast-fed. Risk factors significantly associated with key WHO IYCF indicators in multivariate analyses are shown in Table 4. Older mothers were less likely to have ever breast-fed their child than younger mothers (OR=0·92; 95 % CI 0·86, 0·98). Mothers who had completed higher education had 6·10 (95 % CI 2·41, 15·42) times greater odds of having ever breast-fed their child than mothers who had not completed higher education.

**Table 3:** Key WHO indicators of infant and young child feeding for internally displaced children aged <2 years residing in Kharkiv, Dnipropetrovsk and Zaporizhia *oblasts*, eastern Ukraine, June 2015

Indicator	*n*	*N*	%	95 % CI
Ever breast-fed				
Children born 11 months preceding survey	215	229	93·9	90·0, 96·6
Children born 12–23 months preceding survey	230	248	92·7	88·8, 95·6
Total	445	477	93·3	90·7, 95·2
Early initiation of breast-feeding (within 1 h of birth)				
Children born 11 months preceding survey	140	229	61·1	54·5, 67·5
Children born 12–23 months preceding survey	164	248	66·1	60·0, 72·0
Total	304	477	63·7	59·3, 67·9
Exclusive breast-feeding <6 months				
0–1 month	3	5	60·0	14·7, 94·7
2–3 months	9	23	39·1	19·7, 61·5
4–5 months	5	38	13·2	4·4, 28·1
Total	17	66	25·8	15·8, 38·0
Continued breast-feeding at 1 year (*N*=children aged 12–15 months)	53	99	53·5	43·2, 63·6
Continued breast-feeding at 2 years (*N*=children aged 20–23 months)	13	63	20·6	11·5, 32·7
Introduction of solid, semi-solid or soft foods (*N*=infants aged 6–8 months)	71	72	98·6	88·5, 99·9
Bottle feeding				
0–5 months	43	66	65·1	52·4, 76·5
6–11 months	132	163	81·0	74·1, 86·7
12–23 months	150	248	60·5	54·1, 66·6
Total	325	477	68·1	63·7, 72·3
Minimum meal frequency* (*N*=children aged 6–23 months)				
Breast-feeding	179	186	96·2	92·4, 98·5
Non-breast-feeding	222	225	98·7	96·1, 99·7
Total	401	411	97·6	95·6, 98·8

* Breast-fed children 6–23 months who received solid, semi-solid or soft foods the minimum number of times (two times for infants 6–8 months and three times for children 9–23 months) or more per day and non-breast-fed children 6–23 months who received solid, semi-solid or soft foods or milk feeds four times or more per day.

**Table 4:** Risk factors associated in multivariate analyses with key WHO indicators of infant and young child feeding for internally displaced children aged <2 years residing in Kharkiv, Dnipropetrovsk and Zaporizhia *oblasts*, eastern Ukraine, June 2015*

Outcome	*N*	Predictor	OR	95 % CI	*P* value
Ever breast-fed	477	Mother’s age (years)	0·92	0·86, 0·98	0·017
		Mother completed higher education†	6·10	2·41, 15·42	<0·001
Early initiation of breast-feeding (within 1 h of birth)	477	Two children aged <2 years in household‡	0·29	0·13, 0·67	0·004
		Displaced from Luhansk *oblast* §	0·63	0·42, 0·94	0·025
Exclusive breast-feeding <6 months	66	Female head of household║	0·11	0·02, 0·62	0·012
		Child’s age (months)	0·54	0·32, 0·91	0·021
Continued breast-feeding at 2 years (*N*=children aged 20–23 months)	63	Child’s age (months)	0·38	0·19, 0·77	0·006
Bottle feeding	477	Child’s age (months)	0·94	0·90, 0·97	<0·001
		Mother completed higher education†	0·61	0·40, 0·92	0·018

* No risk factors tested were significantly associated with continued breast-feeding at 1 year, introduction of solid, semi-solid or soft foods, or minimum meal frequency in multivariate models.

† Referent: mother did not complete higher education.

‡ Referent: one child aged <2 years in household.

§ Referent: displaced from Donetsk *oblast*.

║ Referent: male head of household.

### Early breast-feeding initiation

Only 63·7 (95 % CI 59·3, 67·9) % of mothers initiated breast-feeding within the first hour of birth. The prevalence was slightly lower among children born during the conflict (61·1 %; 95 % CI 54·5, 67·5 %) than before the conflict began (66·1 %; 95 % CI 60·0, 72·0 %), although this was not significant (Table 3). Mothers living with two children less than 2 years of age in the household had lower odds of initiating breast-feeding within the first hour of birth compared with mothers living with one child aged less than 2 years in the household (OR=0·29; 95 % CI 0·13, 0·67; Table 4). Mothers displaced from Luhansk *oblast* were less likely to initiate breast-feeding within the first hour of birth than mothers displaced from Donetsk *oblast* (OR=0·63; 95 % CI 0·42, 0·94), with 58·6 % of mothers displaced from Luhansk and 68·2 % of mothers displaced from Donetsk initiating breast-feeding within the first hour of birth (Table 4).

### Exclusive and continued breast-feeding

Exclusive breast-feeding of infants aged less than 6 months was low (25·8 %; 95 % CI 15·8, 38·0 %; Table 3). Of those infants aged <6 months who were ever breast-fed, 19·7 (95 % CI 10·6, 31·8) % were not breast-fed in the day preceding the survey. Younger infants in the less than 6 months age group had a greater odds of being exclusively breast-fed than older infants less than 6 months of age (OR=0·54; 95 % CI 0·32, 0·91). Exclusive breast-feeding of infants aged less than 6 months was less prevalent in households where a female was considered the head of the household (7·1 %) than in households where a male was considered the head of the household (38·2 %; OR=0·11; 95 % CI 0·02, 0·62; Table 4). About half of children (53·5 %; 95 % CI 43·2, 63·6 %) were continuing to be breast-fed at 1 year of age and 20·6 (95 % CI 11·5, 32·7) % of children were continuing to be breast-fed at 2 years of age (Table 3). Older children in the 20–23 month age group had lower odds of being breast-fed at 2 years of age than younger children aged 20–23 months (OR=0·38; 95 % CI 0·19, 0·77; Table 4).

### Bottle-feeding

A high proportion of children aged less than 2 years (68·1 %; 95 % CI 63·7, 72·3 %) were bottle-fed; 65·1 (95 % CI 52·4, 76·5) % of infants aged less than 6 months were bottle-fed (Table 3). Mothers who had completed higher education were less likely to bottle-feed their infants than mothers who had not completed higher education (63·7 and 73·0 % bottle-feeding, respectively; OR=0·61; 95 % CI 0·40, 0·92). Younger children aged less than 2 years had higher odds of being bottle-fed than older children in this age group (OR=0·94; 95 % CI 0·90, 0·97; Table 4).

### Complementary feeding

Almost all infants aged 6–8 months were receiving solid or semi-solid foods (98·6 %; 95 % CI 88·5, 99·9 %) and nearly all children aged 6–23 months met the minimum meal frequency (97·6 %; 95 % CI 95·6, 98·8 %). The prevalence of children meeting the requirements for minimum meal frequency was similar among those children breast-feeding (96·2 %; 95 % CI 92·4, 98·5 %) and those not breast-feeding (98·7 %; 95 % CI 96·1, 99·7 %; Table 3). Most children aged 6–23 months (93·2 %; 95 % CI 90·3, 95·4 %) were given foods from three or more food groups and 84·7 (95 % CI 80·8, 88·0) % received Fe-rich foods (infant formula, meat and fish, and/or commercial infant meat purée) on the day preceding the survey.

**Fig. 1:**
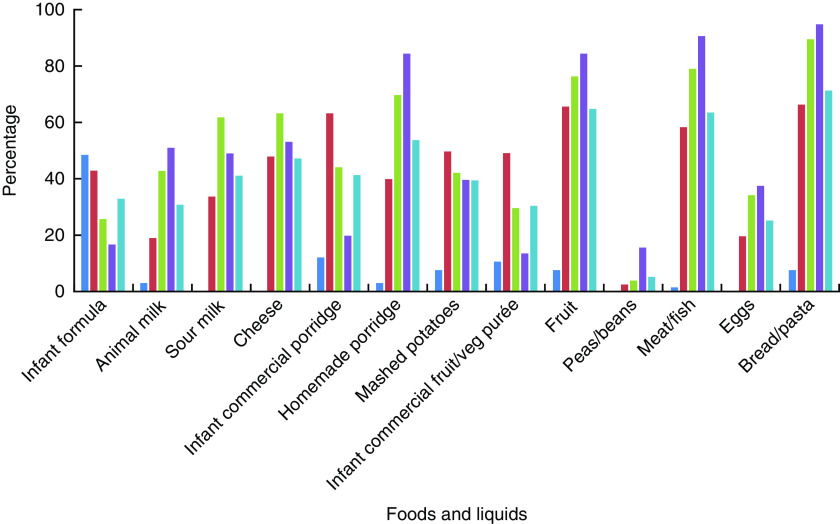
(colour online) Proportion given foods and liquids in the 24 h preceding the survey, by age group (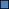, 0–5 months; 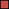, 6–11 months; 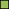, 12–17 months; 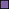, 18–23 months; 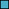, total), among internally displaced children aged <2 years residing in Kharkiv, Dnipropetrovsk and Zaporizhia *oblasts*, eastern Ukraine, June 2015

The proportion of internally displaced children who received various foods and liquids during the day preceding the survey is shown in Fig. 1. Bread or pasta were the foods given to the highest proportion of children aged 6 months or older in the day preceding the survey. A high proportion of children in older age groups (≥6 months) were also given fruit, commercial infant porridge (instant porridge that is marketed to infants and young children) and homemade porridges (prepared from buckwheat, oatmeal, rice, semolina and other grains), and meat and fish. Commercial infant porridge was given to a higher proportion of infants in the 6–11 month age group compared with homemade porridges, which were more commonly given to children older than 1 year of age. Peas and beans were the least frequently consumed food group in all age groups.

**Fig. 2:**
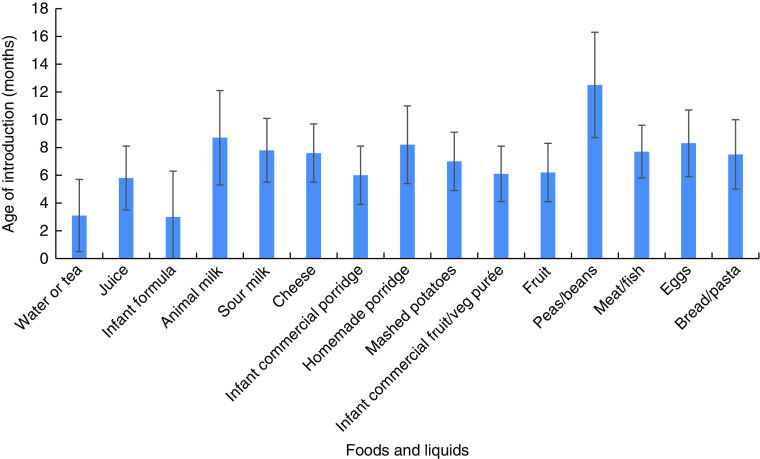
Mean of age of introduction of foods and liquids, with standard deviation represented by vertical bars, to internally displaced children aged <2 years residing in Kharkiv, Dnipropetrovsk and Zaporizhia *oblasts*, eastern Ukraine, June 2015

The mean age of introduction of foods and liquids among children who had already been introduced to those foods or liquids at the time of the survey is shown in Fig. 2. The mean age of water or tea and infant formula introduction was 3·1 (sd 2·6) and 3·0 (sd 2·6) months, respectively. Water was given to the majority of infants aged less than 6 months who were not exclusively breast-fed (85·7 %; 95 % CI 72·8, 94·1 %). Nearly one-fifth (18·2 %; 95 % CI 9·7, 29·6 %) of infants aged less than 6 months received soft, semi-solid or solid foods on the day preceding the survey. Infant commercial porridges, commercial infant fruit and vegetable purées, and whole fruits were the complementary foods with the lowest mean age of introduction (about 6 months of age) and the most common complementary foods given to infants aged less than 6 months.

### Breast-feeding discontinuation

The primary reasons for breast-feeding discontinuation reported by mothers who had stopped breast-feeding prior to survey administration are shown in Table 5. Stress related to the conflict was a primary reason reported by mothers for breast-feeding discontinuation. Mothers who discontinued breast-feeding when their infants were aged less than 6 months were more likely to list stress related to the conflict as the primary reason they discontinued breast-feeding (45·7 %) compared with mothers who discontinued breast-feeding when their children were aged 6–23 months (14·3 %; *P*<0·0001). When mothers were asked their opinions on when it is appropriate to stop breast-feeding, almost half of all mothers (46·9 %; 95 % CI 42·3, 51·6 %) believed they should not breast-feed beyond 12 months of age. Only 29·5 (95 % CI 25·3, 33·9) % of mothers believed they should continue breast-feeding until 2 years of age and beyond.

**Table 5:** Reasons for breast-feeding discontinuation (among those who discontinued breast-feeding prior to survey administration) reported by internally displaced mothers with children aged <2 years residing in Kharkiv, Dnipropetrovsk and Zaporizhia *oblasts*, eastern Ukraine, June 2015

	Breast-feeding discontinuation at <6 months (*N* 105)			Breast-feeding discontinuation at 6–23 months (*N* 105)		
Primary reason for breast-feeding discontinuation	*n*	%	95 % CI	*n*	%	95 % CI
Conflict-related stress	48	45·7	36·0, 55·7	15	14·3	8·2, 22·4
Problems of attachment to breast	10	9·5	4·6, 16·8	13	12·4	6·8, 20·2
Not enough food for mother	10	9·5	4·6, 16·8	4	3·8	1·0, 9·5
Stress unrelated to conflict	3	2·9	0·6, 8·1	4	3·8	1·0, 9·5
Use of bottle for feeding	1	1·0	0·2, 5·2	3	2·9	0·6, 8·1
Work schedule	0	0·0	–	1	0·9	0·2, 5·2
Other	29	27·6	19·3, 37·2	60	57·1	47·1, 66·8
Don’t know	4	3·8	1·0, 9·5	5	4·8	1·6, 10·8

### Acute malnutrition

MUAC was measured in 411 children aged 6–23 months. Mean MUAC was 159·6 (sd 12·1) mm. No child aged 6–23 months had MUAC less than 115 mm and only two children in this age group (0·5 %; 95 % CI 0·1, 1·7 %) were moderately acutely malnourished (had MUAC between 115 mm and 125 mm).

### Humanitarian assistance

Four hundred and forty-seven of the 458 (97·6 %) households surveyed had received some type of humanitarian assistance from non-government sources. The majority of households (87·1 %; 95 % CI 73·1, 81·0 %) received general food assistance. Over two-thirds (70·5 %; 95 % CI 66·1, 74·7 %) of households received baby food assistance. However, only 14·8 (95 % CI 11·7, 18·4) % of households received baby food assistance more than three times and the mean time since last receiving baby food assistance was 2·8 (sd 2·6) months preceding the survey. The most common items received in the most recent baby food assistance package were commercial infant porridge (56·3 %), commercial infant fruit or vegetable purée (49·2 %) and infant formula (44·3 %).

## Discussion

Our study of IDP in eastern Ukraine highlighted several problematic issues related to IYCF practices which need improvement, including a low prevalence of exclusive breast-feeding until 6 months of age, a low prevalence of continued breast-feeding until 2 years of age, introduction of fluids before 6 months of age and a high prevalence of bottle-feeding. These issues were similar to those identified in pre-conflict surveys; however, our study found that stress related to the conflict was a major reason that mothers discontinued breast-feeding^(^ ^14^ ^)^. Our study also found that, overall, complementary feeding in children aged 6 months or older in this population was adequate, as almost all children in this age group were receiving soft, semi-solid or solid foods and meeting their minimum meal frequency. In addition, most children in this age group were receiving Fe-rich foods and meeting the requirements for minimum dietary diversity.

The majority of women had ever breast-fed their child (93·3 %). However, the prevalence of other recommended breast-feeding practices, including exclusive breast-feeding in infants aged less than 6 months, was low. Only about one-quarter of mothers with infants less than 6 months of age were exclusively breast-feeding. These estimates are similar to the MICS 2012 survey data for eastern Ukraine, which reported 96·7 % of children ever breast-fed and 21·3 % of infants less than 6 months of age exclusively breast-fed^(^ ^14^ ^)^. Mothers were less likely to exclusively breast-feed in households where a woman was considered the head of the household, which was similar to findings by Andersson *et al*. during the Bosnian conflict^(^ ^22^ ^)^. These women may have more responsibilities and less time to breast-feed. Similar to our study, Andersson *et al*. found that the proportion of infants who were ever breast-fed was similar before and during the Bosnian conflict^(^ ^22^ ^)^.

Among infants aged less than 6 months who were not exclusively breast-fed, water, tea and infant formula were the liquids most commonly given; this likely reflects common beliefs in Ukraine that infants need water when the mother feels thirsty and it is warm outside. In addition, infant feeding behaviors of mothers were influenced by their social networks (family and friends)^(^ ^23^ ^)^. Some mothers introduced complementary foods before 6 months of age, most commonly commercial infant porridges and commercial infant fruit and vegetable purées. Previous studies have also found women having difficulties adhering to the WHO guidelines of not introducing complementary foods before 6 months of age, often due to outside influences and prior beliefs^(^ ^24^ ^–^ ^26^ ^)^. A study in Australia found that first-time mothers believe that early introduction of complementary foods will help with weight gain and sleeping patterns^(^ ^26^ ^)^. In addition, studies in Australia and the UK found that peer groups influenced a mother’s decision on the timing of complementary food introduction^(^ ^24^ ^,^ ^25^ ^)^.

Contrary to WHO and UNICEF recommendations, a high proportion of mothers (36 %) did not initiate breast-feeding during the first hour after birth. This was similar to pre-conflict data, where 39 % of Ukrainian mothers did not initiate breast-feeding within the first hour of birth and better than in Belarus, where 47 % of women did not initiate breast-feeding during their child’s first hour of life^(^ ^13^ ^,^ ^14^ ^)^. Infants who are not breast-fed within the first hour of birth are less likely to receive colostrum, which contains many protective factors such as antibodies and other immune components^(^ ^27^ ^)^. In addition, infants fed colostrum have lower neonatal and post-neonatal death rates^(^ ^28^ ^)^. Mothers displaced from Luhansk *oblast* were less likely to initiate breast-feeding within the first hour of birth than mothers displaced from Donetsk *oblast*. The reasons for this are unclear; however, if the mothers had given birth prior to displacement, it is possible that health facilities in Donetsk were more supportive of early initiation of breast-feeding than health facilities in Luhansk, which could be an area for targeted IYCF interventions.

A high proportion of children surveyed were fed by a bottle on the day preceding the survey. Bottle-feeding may reduce the mother’s breast milk production and shorten the time to breast-feeding discontinuation, possibly due to nipple confusion^(^ ^29^ ^–^ ^33^ ^)^. Nipple confusion is defined in younger infants as the difficulty for a neonate to establish the necessary latching technique and sucking pattern to extract milk from the breast after exposure to an artificial nipple; and in older infants who have established breast-feeding as the refusal of the breast in preference for the bottle^(^ ^33^ ^,^ ^34^ ^)^. Feeding infants fluids with a cup or spoon instead of a bottle may help to avoid early breast-feeding discontinuation due to nipple confusion and is a better way to provide supplemental feeding to infants^(^ ^33^ ^,^ ^35^ ^)^. Similar to previous studies, more educated mothers were less likely to bottle-feed their infants than mothers with less education^(^ ^36^ ^,^ ^37^ ^)^. These less educated mothers could be a group targeted during IYCF education and counselling interventions.

Fifty-four per cent of mothers were continuing to breast-feed when their child was 1 year of age and about one-fifth were breast-feeding their child at 2 years of age. Almost half of all mothers surveyed believed that mothers should not breast-feed beyond 12 months, despite the WHO and UNICEF recommendations that mothers should breast-feed their children until 2 years of age and beyond^(^ ^11^ ^)^. Stress related to the conflict was the most common perceived reason mothers reported discontinuing breast-feeding before their infant was 6 months old and it was a common reason for breast-feeding discontinuation for all mothers who reported stopping prior to survey administration. Previous studies have found stress to be associated with shorter durations of both continued and exclusive breast-feeding. Dozier *et al*. found financial stress to be associated with earlier breast-feeding discontinuation and traumatic stress to be associated with earlier exclusive breast-feeding discontinuation^(^ ^38^ ^)^. In addition, a study in Australia by Li *et al*. also showed early breast-feeding discontinuation to be significantly associated with stressful life events^(^ ^39^ ^)^.

Almost all children aged 6–23 months were receiving solid or semi-solid foods and minimum meal frequency was met by almost all children. Quality of complementary foods was also adequate, as most children were eating a sufficient variety. The majority of children 6–23 months of age (93 %) were given foods from three or more food groups in the 24 h preceding the survey and 85 % received Fe-rich foods. Children aged 12–23 months received a greater variety of foods than infants aged 6–11 months. In addition, no children aged 6–23 months were identified with severe acute malnutrition (MUAC<115 mm) and only two children aged 6–23 months were identified with moderate acute malnutrition (MUAC=115–124 mm).

The present study is subject to several potential limitations. First, we surveyed only those households included in the registration lists from humanitarian aid agencies, with a working telephone number and available during the survey period. Unregistered households and those without a working telephone number may be receiving less humanitarian aid and/or be of lower socio-economic status, which could have an impact on their IYCF practices. Second, the survey had a high level of non-response among eligible households, likely due to personal safety concerns related to the conflict. In addition, due to security issues, households located in *oblasts* within the conflict zone were not included in the survey. These households may have lower food security than households bordering the conflict zone. In addition, mothers in these areas may also experience greater stress, which may make them more likely to discontinue breast-feeding at earlier ages. Finally, there is the possibility of recall bias in self-reported data.

## Conclusions

In our study of IDP in eastern Ukraine, we identified multiple suboptimal IYCF practices, including non-exclusive breast-feeding until 6 months of age, introduction of fluids before 6 months of age, bottle-feeding and discontinuing breast-feeding before 2 years of age. These were similar to those identified in a broader population in Ukraine prior to the conflict. Overall, complementary feeding in children aged 6 months or older in this population was adequate and most children in this age group were receiving Fe-rich foods and meeting the requirements for minimum dietary diversity. Our study also demonstrated that stress related to the conflict is a primary reason for breast-feeding discontinuation in this population. In order to mitigate the effects of conflict and improve child health, humanitarian action focused on helping mothers cope with stress related to conflict and displacement by supporting maternal mental health and psychosocial support programmes is needed. In addition, humanitarian action should support and encourage women to adhere to recommended IYCF practices if possible and support women for artificial or complementary feeding when adherence is not feasible.
